# In-Flight Hypoxemia in a Tracheostomy-Dependent Infant

**DOI:** 10.1155/2017/3210473

**Published:** 2017-02-28

**Authors:** Jason Quevreaux, Christopher Cropsey

**Affiliations:** Department of Anesthesiology, Educational Affairs, Vanderbilt University Medical Center, 2301 VUH, Nashville, TN 37232-7237, USA

## Abstract

Millions of passengers board commercial flights every year. Healthcare providers are often called upon to treat other passengers during in-flight emergencies. The case presented involves an anesthesia resident treating a tracheostomy-dependent infant who developed hypoxemia on a domestic flight. The patient had an underlying congenital muscular disorder and was mechanically ventilated while at altitude. Although pressurized, cabin barometric pressure while at altitude is less than at sea level. Due to this environment patients with underlying pulmonary or cardiac pathology might not be able to tolerate commercial flight. The Federal Aviation Administration (FAA) has mandated a specific set of medical supplies be present on all domestic flights in addition to legislature protecting “Good Samaritan” providers.

## 1. Introduction

According to the US Department of Transportation, domestic airlines transported 696 million passengers in 2015 [1]. This represented a 5% increase in passengers from 2014. Peterson et al., using flight data taken from 7 million domestic and international flights, found that in-flight medical emergencies resulting in a call to ground-based health providers occurred in 1 in 604 flights [2]. As passenger load is projected to increase in the near future it is reasonable to assume the number of in-flight emergencies will increase as well. The most common causes of in-flight emergency were presyncope/syncope followed by respiratory compromise [2]. Often passengers who are in health-related fields are called upon to render care during these emergencies. The process of flying can be very physically challenging to patients with prior health problems. Passengers often have long walks between terminals or from ground transport, and they frequently have heavy luggage to carry. In addition, they may have limited access to their prescription medicines in-flight. The Aerospace Medical Association recommends preflight assessment of passengers for whom flight might exacerbate underlying pathology. This includes recognizing unstable cardiac, pulmonary, or other disease processes [3]. This however does not preclude the incidence of an acute event.

## 2. Case Report

While traveling as a passenger at a cruising altitude of 36,000 ft, an overhead page went out calling for medical personnel. The author responded to this call. The patient was a 12-month-old male with a tracheostomy tube attached to the portable ventilator his mother had brought on the plane. Also present were a portable suction device, a portable nebulizer, and a portable monitor showing a pulse oximetry reading of 80%. The resident quickly assessed the situation and introduced himself and his qualifications to both the flight attendants present and the patient's family.

A history was gathered from the mother and was significant for an unknown congenital muscular dystrophy rendering poor resting tone and difficulty with self-ventilation. The patient had no known congenital heart issues and no known allergies as per the mother. The child was tracheostomy-dependent and was regularly seen by a pulmonologist. Of note the child was seen by the pulmonologist recently and deemed fit-to-fly. As per the family, because of the muscular disorder the child's lungs were not well functioning, but no additional details could be provided. At home the patient largely tolerated room air without the need for ventilator support. He primarily needed to be mechanically ventilated during nighttime hours or while asleep. His mother reported normal resting oxygen saturations (SpO_2_ > 96%) at baseline. The child was initially on room air unsupported when boarding the plane, but as the plane reached terminal altitude the patient's saturation had deteriorated. The mother placed the child on a pressure support mode without oxygen supplementation. Another responder had attempted to fit one of the airline's portable oxygen cylinders to the machine without success. The mother had attempted an albuterol nebulizer and tracheal suctioning; however, there were no secretions and the oxygen saturation remained low.

Although required to be present on all domestic flights in the United States a stethoscope was unable to be found by the cabin staff; ear-to-chest auscultation and thoracic palpation revealed a symmetrically expanding chest without evidence of obstruction to air movement. The oxygen inflow for the ventilator did not match the outflow from the portable oxygen tanks provided by the airline. Additionally there was not a way to use the auxiliary oxygen supply (the supply providing oxygen to the emergency drop down masks). By utilizing a makeshift design of tape, supplemental oxygen was able to be delivered to the ventilator which resulted in improvement of the SpO_2_ to >90%. As the author was unfamiliar with the type of ventilator the mother had brought, and there was no display to show volumes delivered or returned, he asked the mother to continue to utilize only the settings that she used at home. This was done to minimize the risk of either barotrauma or volutrauma; the mother had been educated by the child's pulmonologist on this specific ventilator and thus had training in its operation.

While initially stabilized, the child began to have desaturation again on descent of the aircraft. The resident checked the tracheostomy tube and realized that the cuff had not been inflated on boarding the flight or on initiation of mechanical support. At this time it was also noted that the child was ventilating with a fair amount of air from his mouth. The cuff was inflated with a few milliliters of air that resulted in an increase in oxygen saturation. However, concordantly with this, palpable crackles began on thoracic excursion. The tracheostomy tube was again suctioned with mild increase in secretions. Oxygen saturations remained >90% throughout this time. The plane landed shortly after and the child was taken to a waiting, on-ground EMS team. The team was informed of the situation and the child was taken to a local hospital for further workup and treatment.

The differential generated included ventilator deficit such as patient fatigue or mucus plugging, negative pressure pulmonary edema, oxygenation deficit due to intrinsic lung disease, resorption atelectasis from high *F*_*i*_O_2_ (during the second desaturation event), intracardiac shunting, thrombotic or embolic event, and malfunctioning equipment. An additional concern would be the decreased partial pressure of oxygen present in a pressurized cabin. The only evidence of poor ventilation came on the final descent so it seemed unlikely that airway obstruction was the primary pathology. More likely it is the hypobaric environment leading to hypoxemia. As stated, thromboembolic events could not be ruled out in this environment. The equipment did not appear to be giving false reading, and the low pulse oximetry reading corresponded to patient's activity level and cyanosis. Because the author was unfamiliar with the particular ventilator he was unable to conclusively determine whether or not it was functioning appropriately.

## 3. Discussion

Hypoxemia occurs at altitude because of decreases in barometric pressure resulting in reduction of partial pressure of oxygen. According to the alveolar gas equation (1), the reduction in the partial pressure of oxygen results in the loss of diffusional capacity of oxygen across the alveolar membrane and endothelium. Internal cabin altitude is the result of cabin pressurization to counteract the flight altitude, that is, the environmental altitude outside of the airplane. The FAA mandates that commercial flights maintain a cabin pressurization equivalent to no more than 8000 ft [4]. This limit allows for a minimum environmental pressure of 564 mmHg, a significant decrease from the 760 mmHg pressure at sea level. The cabin pressurization limit was initially derived based on weight and longevity of pressurization systems, fuel economy, and patient comfort [5]. One study revealed that average cabin altitude across 207 commercial flights was 6341 ft with longer flights resulting in higher average altitudes [6]. Another study revealed an average pressure of 634 mmHg across 45 commercial flights and three airlines in Boeing 747 airplanes [7]. This represents a decrease in the partial pressure of oxygen of greater than 15% from that of sea level.

 Alveolar gas equation:

(1) pAO2=FiO2PATM−pH2O−paCO21−FiO21−RERRER,

where *p*_*A*_O_2_ is the alveolar partial pressure of oxygen, *F*_*i*_O_2_ is the fraction of inspired oxygen, *P*_ATM_ is the atmospheric pressure, *p*H_2_O is the vapor pressure of water, *p*_*a*_CO_2_ is the arterial partial pressure of CO_2_, and RER is the respiratory exchange ratio.

Arterial oxygen tension has been shown to decrease in simulated hypobaric environments more than 20 mmHg in patients with chronic obstructive lung disease [8]. Patients with normal physiology tend to tolerate a hypobaric airplane environment well [9, 10]; however concern exists for patients with existing pulmonary or cardiac or hematological disease and decompensation in this environment [11–15]. Normal respiratory changes to increasing altitude include increased minute ventilation through hyperventilation [16]. Hypoxic pulmonary vasoconstriction increases the resistance through the pulmonary arteriolar and capillary bed with a concordant increase in pulmonary artery pressures [17]. Increased wall tension in the pulmonary vasculature causes an increase in extravascular fluid in the lung parenchyma [18]. Acutely an increase in cardiac output occurs at altitude due to increased sympathetic activity and is largely mediated through increased heart rate [16, 19]. Mixed venous oxygen is expected to drop due to a decrease in oxygen delivery which also drives the increase in cardiac output.

Regarding the patient presented, although not stated by the family, it is likely that the child had underlying pulmonary disease that prevented compensation for the hypobaric environment. It is felt that the hypoxemia originated from decreased diffusional capacity. Due to the decreased arterial saturation resistance in the pulmonary vasculature increased. The tachycardia is likely a response to acute altitude exposure and now decreased right heart output due to increasing pulmonary vascular resistance. This caused or exacerbated right to left shunting resulting in decreasing delivery of oxygen to the tissues. Supplemental oxygen along with mechanical ventilation helped compensate for the hypobaric environment leading to increased arterial oxygen saturations, decreased cyanosis, reduction of heart rate, and increased activity of the child. The second desaturation event on descent of the aircraft is thought to be attributable to developing pulmonary edema. The evidence for this lies in increasing rales in the child's lungs and increasing secretions suctioned from the tracheotomy tube concordant with desaturations on pulse oximetry. Development of pulmonary edema at altitude is secondary to compensatory pulmonary vascular changes [18].

Commercial flights in the United States and international flights with airline agencies originating in the US are federally mandated to have an in-flight emergency medical kit [20] (Table 1). These kits offer a basic first aid kit including supplies for wound dressing as well as more advanced cardiopulmonary resuscitation supplies such as oropharyngeal airways and an automated external defibrillator. The cabin staff are required to have basic CPR certification with recertification every two years [21]. Additionally, some arguments have been raised as to the suitability of both FAA kits for pediatric emergencies [22]. Some airlines have contracted with on-ground medical provider for in-flight emergency advice, even including the use of in-flight telemetry [23–25]. Thus, although every flight should have basic medical equipment available, one of the key initial steps in response to an in-flight medical emergency should be to evaluate the resources at hand. This is especially true for pediatric patients.

One survey taken from physicians in the United Kingdom revealed that the vast majority of survey responders had been called upon to treat patients outside of the clinical setting [26]. Other studies have shown mixed evidence as to the response rate, that is, to treat or not treat, when healthcare providers are placed in this type of situation [26–30]. DiMaggio et al. (1994) found that the two most influential factors associated with healthcare providers responding to a call was distance to the nearest medical facility and familiarity with the type of patient presented [27]. Other concerns were unknown infectious disease or nonwillingness to engage in mouth-to-mouth resuscitation on a stranger [29]. One survey noted a significant increase in response rate when the provider had stronger knowledge of the legal aspects of providing care [30]. Of note on this last point congress passed the FAA Aviation Medical Assistance Act (AMAA) on April 24th, 1998, to protect Good Samaritans on US commercial flight [31]. This law is designed to mitigate legal liability of medically trained persons who respond to in-flight emergencies. The AMAA does not suspend liability if the responder is thought to be in “gross negligence” of care.

## 4. Recommendations

Moving forward several recommendations can be made based on this case and through literature review. The authors of this paper will first echo some broad recommendations of prior articles. Gendreau and Dejohn (2002) published a suggested response to being called to act in the extraclinical setting [23] including introducing oneself to the patient/family, stating qualifications, asking for permission to treat, taking a patient history and physical, and always working within one's qualifications [23]. Chandra and Conry (2013) recommend that seeking input from other healthcare providers present might also facilitate better patient care [32]. Another recommendation from that article is to obtain a personal copy of any forms documenting the incident [32]. The Aerospace Medical Association has published more comprehensive guide for responding providers [33] and would be beneficial to any medical provider with plans for travel. A final generalized recommendation is to gather and evaluate all medical resources available. This includes surveying the in-flight medical kit and discussing with the airline staff supplemental resources such as the ground based telemetry discussed above.

More specifically to the case presented it is recommended that patients with preexisting cardiac and pulmonary conditions be seen by their physicians and discuss fitness-to-fly prior to traveling. If the patient's treating physician is unfamiliar with air travel then consultation with an aviation medicine colleague is advised, specifically if the patient is traveling with medical devices or equipment. Patients with medical devices are recommended to have all the needed components of that device prior to traveling. It is also recommended that the patient or the physician discuss with the manufacturer of the device concerns regarding functionality during flight. The airline should be made aware in advance of the device and all components needed to utilize. This includes discussion regarding supplemental oxygen for O_2_ dependent patients or mechanically supported patients. Also it is important to note, in patients with an endotracheal tube or tracheostomy tube in place, the incidence of mucosal trauma/ischemia secondary to increased cuff pressures. As the barometric pressure drops the air within the cuff expands and increases pressure against the tracheal mucosa. Use of saline in the cuff prior to takeoff has been proposed to mitigate this risk [34–37]. There are drawbacks to this method that should be recognized; saline in the cuff has been associated with elevated cuff pressures at sea level [37] and inflation/deflation with saline increases procedural time versus use of air [34]. Overpressurization of the cuff can be avoided with the use of a manometer [38]. It is also important to recognize that endotracheal tube manufacturers have advocated against this practice [37].

## 5. Summary

Responding to in-flight medical emergencies can be extremely challenging for healthcare workers. In additional to unfamiliarity with the patient and equipment, there are unique physiologic implications due to a decrease in atmospheric pressure. However, this should not serve as an absolute barrier to involvement. In addition, providers should not be dissuaded by concerns for legal ramifications as current law provides a fair amount of protection for those who deliver care in good faith.

## Figures and Tables

**Table 1:** FAA mandated medical supplies on commercial flights [34].

Item	Quantity
*Diagnostic tools*	
Sphygmomanometer	1
Stethoscope	1

*Airway supplies*	
Oropharyngeal airways (various sizes including pediatric)	3
CPR masks (various sizes including pediatric)	3
Self-inflating manual resuscitation device with various mask sizes including pediatric	1

*Basic wound supplies*	
Alcohol sponges	2
1-inch adhesive tape roll	1
Tape scissors pair	1
Tourniquet	1
Nonpermeable gloves pair	1

*IV equipment*	
IV start kit with Y-connector	1
Needles (various gauges)	6
Syringes (various volumes)	4

*Medicine*	
Saline solution 500 cc	1
Analgesic tablet (nonopiate) 325 mg	4
Antihistamine tablet, 25 mg	4
Antihistamine injectable 50 mg ampule	2
Atropine 0.5 mg ampule	2
Aspirin tablet 325 mg	4
Bronchodilator metered dose inhaler	1
Dextrose 50%/50 cc ampule injectable	1
Epinephrine 1 : 1000 1 cc ampule Injectable	2
Epinephrine 1 : 10,000 2 cc ampule injectable	2
Lidocaine 5 cc 20 mg/ml ampule injectable	2
Nitroglycerine tablet 0.4 mg	10
Instructions for use of kit drugs	1
